# Foreign

**Published:** 1841-09-18

**Authors:** 


### FOREIGN.

*Notes on some points of Indian Practice.* By J. MACPHERSON, Assistant Surgeon to the Royal Horse Artillery.—The following rough notes are intended to give some account of the impression which Indian disease has made on me within the first ten months of my residence in Lower Bengal. How far any opinions here expressed may have to be modified by enlarged experience, is impossible to say; but it is right to premise, that my experience has been limited to what has successively come to my notice in our Queen's regiment, and in some hundreds of the Company's Artillery, (both European and native,) the former stationed in Fort William, the latter at Dum Dum, within a short distance from Calcutta.

I believe it is an opinion commonly entertained at home, that the characters of Indian are extremely different from those of European disease—that disease in all cases runs its course very rapidly in this country, and that remedies must always be applied with a very bold hand. This opinion requires to be greatly modified; and with the exception of those formidable diseases, cholera, dysentery, and hepatitis, no others appear to call for especially active treatment, as it is termed. Many practitioners, however, think very differently, and deplete and administer calomel in quantities, often followed by the most disastrous consequences; and there is certainly in this country a much greater risk of too much being done for a patient than too little.

After all there is a great deal of uniformity in Indian disease, as well as in our treatment of it. The last of these facts is, in a considerable degree, to be attributed to the writings of the late Mr. Twining, whose book is usually adopted by assistant surgeons, on their first arrival, as a hand-book. In this there is certainly one advantage, that by uniformity of treatment they are soon enabled to form a fair estimate of the value of the remedies which they employ. In a country so vast as India, it must be a matter of regret, that so little has been done to develope the resources of its materia medica, for we still depend wholly on England for our medical supplies; and the partial substitution of chirayta for gentian is almost a solitary instance of a native being introduced with success in the place of an European remedy.* In the following notes, which possess no novelty, and chiefly refer to a few points of practice among Europeans, it is not proposed to enter into any details regarding diseases whose history and pathology are well known, and have been carefully described.

### *Diseases of Europeans.*

*Fevers.*—There appears to be nothing peculiar in the character of intermittents in this country; purgatives, occasional general and local depletion, (the last of which should be employed with much care,) quinine; and in obstinate cases, the liquor potas. arsenit. (the judicious use of which is scarcely ever followed by any disagreeable consequences,) are our main remedies. Mackintosh's plan of bleeding in the cold stage, a most dangerous one ac-

* Much is however expected, and with good reason, from Dr. O'Shaughnessy, who has been for some time engaged in preparing a work on this subject.

cording to all analogy, has met with few advocates and seems to be almost forgotten. Narcotine, lately introduced into practice by Dr. O'Shaughnessy, although it may possess some febrifuge virtue, is undoubtedly very far inferior to quinine. Agues, of course, bear in all countries a local character; and the Arracan fever has almost the inveteracy of that of Walcheren. I shall say little of *remittents*, as my experience of them has been very small; they are very insidious in their character, and demand constant observation on the part of the physician. Slight cases have recovered under the use of leeches, saline purgatives, and antimonial diaphoretics, followed sometimes by the moderate exhibition of quinine.

*Continued fever*, is not of common occurrence, and petechiæ, or typhoid symptoms, are rarely seen. Patients, however, not unfrequently fall into a very low state, attended with cerebral symptoms, in which cases stimulants and local counter-irritants are indicated. It may be here not unworthy of remark, that in a few fatal fever cases, which were examined last summer, no abdominal lesions could be detected. Slight effusion into the ventricles, and slight congestion of the vessels of the brain and arachnoid were the only perceptible morbid changes.

There was a slight form of fever very prevalent in June of last year, which may be termed *catarrhal*. As many as eighteen men have been admitted into the hospital in one day, all complaining of headache, and of pain in the loins, with their conjunctivæ injected, and general irritation of the mucous membranes. A few leeches with saline purgatives, always effected a cure.

Some account of the *Chusan fever*, as I have heard it described by Mr. Wrightson, and other gentlemen who have had these cases under their care, will probably be interesting, both from the peculiarity of its features, and from the fearful ravages which it has made among some of our corps, especially the Cameronians. The disease usually commences with an attack of fever, which is followed by dysentery, sometimes of a pretty acute character. To this usually succeed a chronic diarrhœa, and a general wasting of the system, to which we may add occasional anasarca and ascites. The tongue assumes a morbidly red appearance, looking as if the epithelium had been removed; yet the appetite remains good. From this condition the patient rarely ever rallies, an increase of diarrhœa supervenes, and he is carried off in a few days. The post mortem appearances are: the stomach and intestines pale and shrivelled, not ulcerated, (though some think they have detected slight abrasions near the pyloric orifice;) the liver and spleen are generally enlarged, soft, and gorged; and, what appears to be most characteristic of this disease, the mesenteric glands are enlarged to the size of beans, and full of concretions: so that mesenteric obstruction, and consequent atrophy, would almost seem to be the cause of death.

The symptoms and appearances above detailed were common to the Europeans, and to the Bengal volunteers; and it is a melancholy fact, that scarcely a man of the invalids, who have returned to this country, is likely to recover. No very satisfactory reasons have hitherto been assigned for the origin of this disease, although the report of the superintending surgeon may, when published, throw some light on the subject.

Of *cholera*, which has prevailed pretty extensively during the last month among Europeans and natives, it need only be remarked, that the usual treatment is, to exhibit calomel in ten grain and scruple doses, combined with one grain of opium, every hour or two hours, according to circumstances; to give stimulants internally; and externally to use frictions and sinapisms: and very unsatisfactory this treatment often is. One point regarding the prognosis, lately suggested by Dr. Mouat of her Majesty's service, may be worthy of attention. He states that when pulsation of the heart cannot be detected with the stethoscope, the case is hopeless; that if it can, the patient may recover. The last epidemic of cholera in Bengal occurred some months ago, in the decayed city of Dacca, in which most of the inhabitants are in a state of abject poverty.

*Syphilis* is very prevalent both among Europeans and natives; but it does not seem to present any peculiarity worthy of notice. Secondary symptoms are not very common, and periostitis occurs only in cases in which the use of calomel has been excessive. I have not yet met with any case of syphilitic iritis. The majority of cases get well readily, under the use of saline purgatives, and of low diet; and sarsaparilla combined with bichloride of mercury, or with iodide of potassium, are the most useful auxiliaries in secondary cases.

*Delirium tremens* is unfortunately a very common affection, usually occurring, as experience shows, among confirmed tipplers. Men who are in the constant habit of drinking, though not in sufficient quantities to produce intoxication, are much more liable to attacks of it than those who only indulge occasionally to excess. In these cases depletion is not often indicated. The moderate use of opiates, along with purgatives, appears to offer the most successful mode of treatment. Terebinthinate enemas, and opium and camphor in the solid form, generally produce the very best effects. Cannabim, the active principle of hemp, as lately introduced to notice by Dr. O'Shaughnessy, has been employed with some advantage in this disease; but its efficacy is understood to be much less than that of opium or morphia.

The form in which *rheumatism* usually occurs is frequently very obstinate; it is oftener chronic than acute; and I have not hitherto met with any case of rheumatic pericarditis. Diaphoretics, guaiacum, and small doses of iodide of potassium, are useful in its milder, and calomel and opium in its severer forms.—Where there is thickening about the joints, the local application of the tincture of iodine is sometimes very efficacious, as also occasionlly in indolent buboes.

*Dysentery* is one of the most formidable diseases with which we have to contend, whether the violence of its attacks, or the frequency with which relapses occur, be considered. In its acuter forms I have not seen a great deal of it; whereas, in its milder ones, where the boundary between it and diarrhœa is indistinctly marked, cases of it present themselves daily. It cannot admit of a doubt that calomel and drastic purgatives have been most injudiciously used in this disease, and that a return to a milder mode of treatment will be attended with the most beneficial results. Indeed, it has been stated, that the present distinguished Inspector General proposes to issue an order, forbidding the use of calomel among the Queen's troops. There is no difference of opinion as to the propriety of free depletion in the earlier stages of this disease, followed up by the use of mild purgatives, among which castor oil is quite invaluable. The combination of blue pill, ipecacuanha, gentian, and hyosciamus, so commonly employed, is a most useful preparation; and opium is also a very important remedy, although the belief that it merely masks the disease is very prevalent. An opiate enema, or Dover's powder, may in most stages of the disease be most usefully administered.

On post-mortem examination the colon is always found to be the chief seat of disease. In one case it was enormously ulcerated and thickened near the caput cœcum; while in another it was studded with patches of deep, though comparatively superficial ulceration.

*Hepatitis*, in its different forms, is of course a very common disease, although acute abscess appears to be less common here than at Madras. Last year the number of cases of hepatic abscesses was very considerable, and their progress was as usual very insidious. I have seen a man admitted into hospital with an anxious countenance, and complaining of pain in his right side, who died suddenly two days after, in which case the whole of the right lobe was found converted into one huge abscess. In this man the abscess of his liver must have existed for many days before his admission, although it had caused him only a little uneasiness. The late universally respected Inspector General* was ill for three months before his death. The chief symptoms were general languor and debility, along with irritability of stomach. He was attended by the most eminent physicians in Calcutta, who frequently examined the region of the liver, in which nothing could be detected, and uneasiness was never felt; yet he was suddenly seized with pain in his side, expectorated a large quantity of pus, and sunk; dying, no doubt, of hepatic abscess.

* Dr. Donald Macleod.

I remember another case, in which the diagnosis was very difficult. A young man had a constant fixed pain in his right side, in the region of the liver, and in his shoulder, accompanied with a dry cough; in consequence of this his case was at first treated as an hepatic affection, and abscess of the liver was suspected. Ultimately the case became evidently one of phthisis; and on *sectio cadaveris* a large vomica was found near the bottom of the right lung, the apex being nearly healthy. The liver did, however, bear some traces of former inflammation.

Another case attracted a good deal of attention. The health of an hospital serjeant had long been much impaired, when he was attacked with a constant diarrhœa; his motions being of an excessively unhealthy character, and protrusion of the rectum followed. A palliative treatment was adopted, but the symptoms got worse: the abdomen excessively attenuated. On laying the hand on the abdomen, what appeared to be a pulsating tumour was felt, and he was generally believed to labour under aneurism of the aorta. He gradually sunk. The aorta was carefully examined after death, by Mr. Freeman, and found perfectly healthy; while the liver was studded with abscesses, and the small intestines partially ulcerated.

There appears to be but one opinion as to the treatment of acute hepatitis; and free depletion with the use of calomel is the almost invariable practice. When the case has once advanced to the formation of abscess, the treatment is very difficult; but I have not met with any case in which the abscess opened externally, or which had its contents evacuated by artificial means.

Affections of the lungs are not among the most common cases, as soldiers before coming to this country have usually passed the age when tubercles are most likely to develope themselves; yet phthisis occasionally appears, and runs its course as at home. Chronic bronchitis is by no means of unfrequent occurrence; and in a climate where the changes of temperature are so rapid, common catarrhs are frequent. I have not seen many affections of the heart, though inordinate action of it, and palpitation, sometimes attend convalescence from fever, and often prove rather intractable.

The diseases of children are, if possible, of more importance here than at home; and it is quite extraordinary how rapidly a diarrhœa, or cephalic attack, during the progress of dentition, will carry off its victim. I have seen one well-marked case of cholera in a child of seven, who died a quarter of an hour after its admission into the hospital. A detachment which lately arrived from N. S. Wales lost several children of dysentery, and in every case lumbrici were present. Nothing peculiar appears to be indicated in the treatment of these affections. No medicine exceeds in value the Hydrarg. c. creta, combined with other remedies; yet it is a common saying, that children will slip through one's fingers in spite of the most judicious treatment.

*Surgery.*

There can, of course, be little that is peculiar to the surgical diseases of this country; and this will immediately strike any one who peruses Mr. Brett's recent work on the Surgical Diseases of India. The cases among Europeans are usually of the most trifling nature, and operations are very rare. Acute otitis and otorrhœa are at times very prevalent.

I remember one case in which a horse artillery man, who fell backwards from his horse, came into hospital with both humeri dislocated under the clavicle.* In another case of a native who had dislocated both condyles of his lower jaw by yawning, every effort at reduction was unavailing; yet he possessed the powers of mastication and of speaking to a considerable degree, and, as the jaw had then only been one month dislocated, it would no doubt in time become servicable enough. The difference between Europeans and natives, in the power of sustaining any great injury, is very marked. Thus I have, within the last few days, seen a native labouring under extravasation of urine of thirty-six hours standing, and who exhibits little constitutional disturbance; while the parts would have ere now been sloughing in a European, and the patient labouring under violent irritative fever.

From this feature in the constitutional power of the natives, operations may be undertaken on them which would be out of the question in the case of Europeans; and there is a wide field for operative practice, on cases of stone, enlargement of the scrotum, and cataract. Some forms of ophthalmia in this country take on a very intractable character. In one case, occurring in a European, the inflammation was limited to the conjunctiva, and was of a remittent character. The eye would be nearly well in the morning, and before night highly injected. Every variety of alteratives, as well as local applications, was made use of without any permanent benefit. In another case, in a half caste, the sclerotica and cornea of both eyes were involved, and the inflammation was at times so violent as to threaten the destruction of the corneæ. Those more violent attacks were moderated by the use of calomel and opium; but it has been found quite impossible to get the eyes well, and they are rather worse now than when he came under treatment four months ago.

*Diseases of Natives.*

Some of the most common diseases of natives are ague, affections of the spleen, dysentery, rheumatism, lepra, and psoriasis; and among these, disease of the spleen offers the chief peculiarities. It is always attended with general debility, and especially of the vascular system, from which some have been led to suggest an analogy between it and scurvy.

Enlargement of the spleen is usually treated with a combination of tonics and purgatives—of iron and gentian, with colocynth and scammony, &c., but with very small success. More confidence might perhaps be placed, with justice, in the exhibition of quinine, and the mineral acids, or even the liquor arsenicalis. Some have proposed the use of iodine, but spleen patients are seldom in a state in which they could bear its exhibition. It is a common saying, that practice among natives is not satisfactory; and one reason, though it does not say much for our humanity, is, that we often do not take enough of interest in our patients: another difficulty always meets us, in the impossibility of regulating their diet. And here, it may not be out of place to mention one interesting fact, which shows how much that form of functional amaurosis, termed day blindness, depends on the condition of the digestive organs. In the Burmah war some of the troops suffered much from this affection, until it was discovered that they had a deficient supply of the condiments usually employed by them in making their curries. Whenever this deficiency was supplied, they all recovered perfectly.

The subject of medical and of general statistics appears to be daily exciting more interest at home, and the reports lately compiled from the records in the Director General's office are replete with valuable information. Some cultivators of statistics, however, appear to indulge in many visionary ideas, and even talk of reducing the principle of the science (far better termed art) of medicine to the accuracy of mathematics. In all this they seem quite to forget that disease is a far more varied and complicated problem than any one in the whole range of the physical sciences; and that every thing connected with its causation is hid in far deeper mystery than the principles of meteorology, of which, after continued and accurate information (far more accurate, be it remembered, than can ever be applied to disease) we are only beginning to recognise a few outlines. It is, nevertheless, much to be regretted that the medical authorities in this country have paid so little attention to the statistics of disease. Such records as do exist among the company's troops are very imperfect, and relate chiefly to Europeans. Those among the Queen's troops are more perfect, but still leave

* He afterwards returned with one of them dislocated again. I easily reduced it with my hand.

much to be desired. One very considerable evil arises from the very defective nature of the classification of disease adopted in the returns among the company's troops; and it will scarcely be credited that in a new one, recently adopted by the Medical Board, cataract has gravely been set down among nervous diseases!

As some of the readers of the Gazette may take an interest in the state of our periodical literature, I subjoin a list of the scientific and medical journals at present published in Calcutta. The Asiatic Society continues to publish its journal, and the Medical and Physical Society publishes occasionally the papers or abstracts of the papers read before it. Mr. Corbyn's Scientific and Medical Journal both go on, and Mr. M'Clelland has lately started a journal of Zoology and Natural History, which is likely to be supported with spirit.

*Dum Dum*, March 8, 1841.

P. S. Farcy and glanders have been very prevalent among the troop horses here for some years, usually attacking the animals in the rainy season. Besides tubercular deposits in the lungs, abscess and softening of the ribs are often found after death. No case of glanders in the human subject has hitherto occurred here.

*Lond. Med. Gaz.*

---

*New Treatment of Hydrocele.* By M. Jobert.—This mode of treatment, which is founded on the same principles as that proposed by M. Velpeau for the cure of inguinal hernia, has already been put in practice by M. Jobert in several cases, and with every appearance of success. The following are the steps of the operation, as described in the report of the first case in which M. Jobert had followed the practice.

A small and very narrow bistoury was introduced at the middle and anterior part of the tumour, its cutting edge being directed inwards, and its back outwards. When the tunica vaginalis was pierced, M. Jobert depressed the handle of the bistoury, and carried it on in a direction parallel with the cord. Having reached with its point the summit of the tumour, he turned the cutting edge forwards as if to incise the integuments. This done, he withdrew the bistoury, dividing with its point the tunica vaginalis from the upper end of the sac to the point where the skin had been punctured. The bistoury was again immediately introduced by the same puncture, and the inferior part of the tunica vaginalis incised in the same manner. The fluid was then evacuated by the small puncture and compresses soaked in a solution of muriate of ammonia were applied. The patient suffered little during the operation, and nothing afterwards.

The day after the operation a small longitudinal depression was felt through the scrotum, corresponding with the point where the tunica vaginalis had been divided.

The operation was performed on the 22d of June, 1840, and the patient left the hospital about the middle of July, to all appearance cured.

In a case on which M. Jobert has since operated, in addition to the longitudinal incision he made likewise a transverse one, with the view of giving greater certainty of success.—*Edinburgh Monthly Journal of Medical Science.*

---

*On Vaccination in India.*—The following facts are from notes which accidentally escaped the destruction of many other papers, and were collected between the years 1825 and 1829, while I was vaccinator (or superintendent of vaccination) over a very extensive territory. My operations were, however, limited to the province between Bombay and the government dominion, and the mountain range and the sea; for I found ample occupation in this, the Southern Konkem. The numbers vaccinated varied; latterly it was above thirty thousand in the year. In fact, it became so great, that there was no limit to the number that could then be vaccinated, except the two natural ones—of the amount of means, and the number protected from variola. My establishment consisted of four native vaccinators, four apprentices or juniors, and six peans. The duty of the latter was to attend to the instructions of the vaccinators and apprentices qualified to act by themselves, to give notice of vaccinating days to the villages, and assist in collecting the children. No compulsion was permitted. At first I found the Brahmins very generally opposed to it, affirming that it was not in accordance with their religious tenets, and the Wanees, or higher merchant class, followed in their wake. The Moussulmins again opposed it on the grounds of fatalism; but being constantly amongst the people, reasoning with them, and showing the good effect resulting from the operation, their prejudices gradually gave way, and the Brahmins finally became amongst the most eager to accept the proffered boon.

Indeed, and it may show how, in a brief space of time, religious prejudice even may be placed in abeyance. When I first began my wanderings amongst them, if I touched a Brahminee child, the child, and also the person holding it, if it were in arms, required ablution; and long before I quitted the appointment, I have often been wearied with the numbers of these children put into my arms for good luck.

The appointment of four vaccinators, with an establishment as above mentioned, was made, I think, in 1822 or 1823, by that talented and excellent man, the Honourable Mountstuart Elphinstone, then the enlightened governor of Bombay. In 1824, it was made a condition of holding the situation, that the medical officer (by rank an assistant surgeon) should be a linguist in one, at least, of the native languages. The districts were far too large, (I never was able to attend to above a third of mine;) but if the success of the experiment was in other divisions at all commensurate with that in the Southern Konkem, it has seemed to me that only two reasons could be advanced for not increasing very largely the number of appointments; the one financial, the other the paucity of medical officers in the establishment. The scale of remuneratiou fixed by Mr. Elphinstone's government was liberal, but not too high, for the appointment was a very laborious one; and in a country where there are no places of accommodation, as inns, &c., involved great exposure, and was attended by very considerable expense. The salary of 600 rupees, six hundred per mensem, was reduced in 1829 to 350 rupees; to which is to be added subsistence money, a net pay of two rupees per diem.

Some inquiry was attempted now some years ago in Bombay, relative to vaccination, and I handed in these notes, as conveying something new, to add to the mass already known; the expected information, however, was not obtained, and these were laid on the shelf. My last batch of periodicals gives me a review of the report of the vaccination section of the Provincial Medical Association; and as there are some facts in these notes which are not noticed there, and seem not known to the profession, I have got them returned, and now transmit them to you. I remain, sir, your obedient servant,

ALEXANDER DUNCAN, Surgeon.
Dapoolie, Bombay Presidency, April 14, 1841.

P. S. When I was at the neighbouring hills, I was informed that there was difficulty in producing vaccine there, and that the vesicle was very generally smaller than in the low country, and with a more confined variola. It was so in one of my children. Is this the case in other elevated regions, as Switzerland? I hope some of your readers will be able to inform us of this. The three principal stations in this finest climate in the world, are Potacamand, Kotagharry, and Connoor, at altitudes respectively of about 7,400, 6,570, and 5,886 feet.

First. Vaccination varies no less than three days in the time required for the perfecting of its vesicle. It may be a day in advance of its proper period, or one or two in retardation. I have been at considerable pains to ascertain these points fully. In the first case there could be no doubt of accuracy; there was the vesicle perfect before its time. In the second case, although it might be accounted for by a supposed retardation of absorption of the virus, still that this was not always the case was manifest by its occurrence in whole families, as brothers and sisters, or as grandmothers and their descendants, (in one case including the great-grandmother,) vaccinated at the same time and place as hundreds of others,—thus showing it to be constitutional and hereditary. The retardation is more frequent than the acceleration, and of one than of two days. The lymph from either produces in others the usual phenomena.

Second. Vaccinia and variola are compatible with each other, until the period of maturation of either, and then the other decays along with it. Thus, the contagion of small-pox may have been imbibed for days prior to vaccination being practised, and yet this latter may amount to a full security against its principal evils. After I had clearly discovered this law, had seen the variolous eruption proceeding and the vaccine progressing, in several cases, each disease unaffected by the other, until the conclusion of the eighth or beginning of the ninth day, of the latter I was then able to give a favourable prognosis, even in severe confluent cases; i. e., when the vaccine was in such time that its eighth day should supervene ere the desiccation of the variola should commence. The progress of the small-pox was invariably arrested in such case, and the eruption died away, like any other cutaneous disease that had sustained a cure; and there was either no fever, or a very slight one, arising from the extensive irritation the system had previously suffered. It had, in fact, lost its specific character of variola, and all danger was over.

I witnessed this result in a considerable number of cases. In a young Brahminee, about sixteen or eighteen, the variola was a severe confluent, and her parents and friends, and even a skilful vaccinator, were fully persuaded she must die; as five of her brothers and sisters, and a mother and her two children, had recently done in this same house. I pointed out in the midst of the variola on the arms, the vaccine vesicles distinctly pitted in their centres, and told them these would save her; that the symptoms would be as those they had seen in the others till the end of the eighth day from vaccination, or beginning of the ninth, and that from that time she would recover; and, to their astonishment, it was so. Her face was tender, and scarred at first; but some months afterwards a very great improvement had taken place, and I was not without hope that the scarring would, in time, entirely disappear.

*Failure with Dry Lymph.*—I have no doubt that failures from the use of dried lymph are very generally owing to the minute quantity laid on the glass, or point. The quantity that might be amply sufficient for the production of several vesicles, if used in its fresh liquid state, may not, when dried up, be sufficient for one. For, first, it is next to impossible to introduce the whole of it. Secondly, it may not be so immediately or completely absorbed.

Thirdly, a portion, though not the whole of it, may be deteriorated and inert; and hence there may not be a sufficiency for the production of even one vesicle, whereas, if a larger quantity were laid down, there would be more to work upon, and a better chance of success. This I know, in an extensive experience, that I failed very frequently with the small, and very rarely with the large quantity.

As to the best vehicle for softening and diluting the dried lymph, (or the scab,) whether water or milk, and whether cold, hot, or tepid, I do not think even this has been sufficiently tested. I am of opinion that tepid is the best state, and milk the better vehicle.

It is said by able physiologists, that the cuticle should be slightly raised in vaccinating, but no blood ever be drawn. Here is theory, but it is at variance with the success of practice. The most expert vaccinators I had ever seen, both in speed of operation and success in result, were the four senior natives in employment where I was appointed superintendent. I had tried my hand on a good many hundreds before, but these men beat me out and out; so I set to work and soon equalled them. I have frequently timed ourselves, and including delays for the flow of lymph, and opening new vesicles, not hurrying, but working deliberately, we made six and a half punctures in the minute. Did we avoid drawing blood? Assuredly not. We did not wish to see it stream, but we liked to see a drop or two; and this I know, that the experiment with the cuticle only slightly raised, or the cutis very slightly penetrated, were not attended with that success in our hands that the deeper puncture was. It seems to have escaped attention that this deeper puncture cuts more absorbents, and passes the mouths of more also than the more superficial, and thus affords a better chance of immediate absorption. Who ever has advanced the opinion that a very slight puncture by the poisonous fang of a serpent is of necessity more deadly than a deep one? Just so in this; should the blood flow freely, it may wash away the matter. It does not always, but it may; but never mind its appearing; a moderately deep puncture affords a better chance of success, but not a straight downward one; it should be oblique, or sloping.

There is a tact in vaccinating. No new hand had the same success as the old. The new took more time, were at much more pains, but for all this they failed much more frequently. We usually made three punctures with one charge, keeping the point of the lancet sloping downwards. A superintending surgeon denied the possibility (and he had vaccinated largely himself—here, again, was theory—for he had not tried it) of raising three vesicles with one charge. I showed it to him in practice. We rarely failed. Sometimes, indeed, only two came forward, and at times the system would not take the disease. It was usual to make three punctures in each arm of those above six months old, and four in grown-up people. Whenever the arm was done, pretty strong pressure rapidly over each puncture with the shoulder of the lancet was made; this certainly tended to repress bleeding, and probably to promote absorption.

There is a beauty in the vaccine vesicle, *of degree*. Take a hundred arms, with their complement of vesicles, and all perfect; out of these, some few, say ten or twelve, will be more beautiful than the others; and compare these, also, with each other, and a few will be found more beautiful than the rest. I would select the most beautiful to vaccinate from, and yet I know not if it be of any consequence. In taking the lymph, the lancet was entered at the base of the vesicle, and worked so as to cut the cells all round at the base, but leaving the cuticle entire, except where the lancet entered. The first lymph had usually a little blood with it, and was put aside; then it came quite pure, and continued to flow for some time. We had to wait now and then a minute or so to give it time. It goes on secreting very rapidly when thus treated; and this secretion is proved by its effects to be equally efficacious with the lymph that was contained in the vesicle when first opened.

Vaccinating from 1,200 to 3,600 in the month, in a rugged country of rock, and hill, and ravine, and petty villages, it was out of the question that separate and minute instructions could be given to the individuals. General rules were therefore given, and these were generally, certainly, if not always, attended to. Thus, as it is well the arm, when vaccinated, should not be washed for a day or two, nor rubbed nor bruised for the whole period; and as a favourite mode of drying children is to seize them by the arms and swing them round, so the rule was laid down that no ablution of a vaccinated person should be made for eight days. The rule was attended to; it came to be thought a necessary thing, and I know no evil that arose from it, but am certain that in very many cases it did good, by saving the arms from being bruised.

*Modifying Causes.*—It has not appeared to me that the season has any decided effect on the vaccine, but there are circumstances which have. If the person from whom the lymph is to be taken has travelled so as to be suffering from fatigue, or is oppressed by the heat of the day, or by hunger, or by fear, either total failure may occur, or the result may be an impure disease from a pure one, (i. e., pure till that moment.) I do not say the result will always be such, I know it is not so; but I have seen enough to know there is a risk, and a considerable one, too; I except that sort of dread that is manifested by kicking and roaring, unless very long-continued. But everything that exhausts the cerebral energy, has seemed to me occasionally to produce a deterioration of the vaccine secretion. It is a subject worthy of the fullest investigation. Perhaps many of the failures so often occurring may be traceable to this source; i. e., from a perfect vesicle lymph may have been used, taken off under circumstances of cerebral distress, and give rise to spurious disease. One day I rode into a small village, and found the whole of the vaccinated with spurious vesicles. I went into the neighbouring one, and here all were perfect; they were vaccinated on the same day from one child. The vaccinator was pressed for time, and, having finished the latter, hasted away, in the heat of the day, and without giving the child time to rest and to take food, to the other.

*Efficacy.*—The efficacy of vaccination in the Southern Konkem was perfectly satisfactory. When small-pox appeared in any village, and the vaccine was sent thither and fully established, then the former was completely arrested. Of this the people were latterly fully aware. In the Sawunt territory, the Muharatta doctor of a large straggling town ridiculed the idea of our preventing the contagious variola by our process, and said that he would show us how to produce the proper preventive disease. I desired him to wait for two months after I was done with the place. He then inoculated above two hundred, of whom some had swollen arms in consequence, and some had an eruption of miliary-looking vesicles, chiefly in the arms; but not one had small-pox, or anything like it. The man very feelingly remonstrated with me, that I had taken the bread out of his mouth; for if small-pox had broken out in that town,* then most of those, in whom we *had put our charm*, would have been brought to him, and inoculated at from half a rupee to three rupees, and sometimes five or more each.

Inoculation of small-pox was extensively practised in all the Southern Konkem. In the northern and central parts the common potters were the performers; and hence the inoculated went by the name of potters' pock; in the southern part I found the common doctors perform it; it is most clumsily but efficaciously done. A portion of skin below the elbow on the palmar aspect is abraded with a piece of broken earthenware, and then a small quantity of cotton, well imbued with the virus, is tied on firmly. It very frequently leaves a large permanent scar.

This practice must be of considerable antiquity, and may have originated amongst themselves. But that is conjecture. I was unable to trace it to any date or source; but it is managed with great practical good sense, such, indeed, as was never in force in our own country; *for it is not permitted to be done in any village until small-pox has first appeared therein;* and hence the disease is not carried the length and the breadth of the country by inoculation, as it too often was with us; and they are very strict in preventing communication with infected villages. Such, indeed, are placed in quarantine—a quarantine that is founded on opinion, and very rarely, I am confident, knowingly transgressed. The disapprobation that would universally fall on the transgressor, would render his life irksome to him. I was once solicited to get a man hanged, as the English put people, even Brahmins, to death in that way for murder, for an infraction of the rule. Serious, indeed, were its consequences. He had unwillingly (I believe) come in travelling into an infected village and slept there, and then came into a Brahmin's in another; it was one of those houses, or accumulation of houses under one roof, where many families dwell. Five young persons of one family, and the mother and two children of another, fell victims to the disease at its outset. The complainant, the widower, and near relative of the others, urged that his guest's conduct had been as criminal as that of a slayer of men, and pathetically mourned the havoc that ensued from it.

I mention this merely to show the strong opinion that is held on the subject; of course I could give no redress, nor inflict any punishment. The spread of the disease was speedily arrested by the rapid and extensive introduction of the vaccine; and here I had several opportunities of observing the fact already mentioned, of the two diseases proceeding simultaneously till about the close of the eighth day of the vaccine, when both begin to cease. It was here that the confluent case occurred in the Brahminee young lady, mentioned above.

I may mention, that it is of some consequence in vaccinating that the touch should be perfectly sharp; failures are more fequent when it is not; even those sharpened at our own stones do not answer perfectly; they are sharp, but not so smooth as out of the hands of the makers; they do not last well, and they soon rust. It would seem that any roughness in the instrument the poisoned wound is made with, is against the absorption of the poison; the best for the coast are Stoddarts' Wootz, or Indian steel lancets; each of them is worth at least three or four of any other kind; they are so hard that they keep their edge long, and they do not rust near so rapidly. This steel was imitated, but the imitation was faulty; I detected it in use, and made the Stoddarts acquainted with the fact, i. e., of its not being the same, and not at all so good. It would seem, they supposed they could form an equal compound to the original by combining the various ingredients which chemistry had discovered it to consist of; as this was not the case, they, of course, were to abide by the original. The silver steel does not answer at all well on

* I had him instructed in vaccination, and informed him where he could always get a supply of vaccine, if he by chance had it not.

this coast; it blackens and rusts very easily.

P. S. It being now proved that vaccinia is only variola, modified by passing through the genus taurus, perhaps, also, the transit into a milder and more rapidly progressing disease, may not the explanation of the two running their course together for a time be, that as the modified runs its course so much more speedily than the original, so, having once taken effect, its preventive power is shown in destroying the virulence of the original at the time of its becoming perfect?

*Vaccinated in* 1828 *in the Southern Konkem.*

| | Males. | Females. | Total. |
|---|---|---|---|
| January, | 1430 | 1374 | 2804 |
| February, | 1348 | 1205 | 2553 |
| March, | 874 | 875 | 1749 |
| April, | 2196 | 1039 | 3235 |
| May, | 1264 | 1033 | 2296 |
| June, | 813 | 822 | 1635 |
| July, | 642 | 578 | 1220 |
| August, | 1201 | 1150 | 2351 |
| September, | 1635 | 1531 | 3166 |
| October, | 1774 | 1549 | 3323 |
| November, | 1839 | 1745 | 3634 |
| December, | 1860 | 1756 | 3616 |
| | 16,876 | 14,706 | 31,582 |

*London Lancet.*

*Cases and Observations on the Molluscum Contagiosum of Bateman, with an account of the minute structure of the Tumours.* By Robert Paterson, M. D. &c. Physician to the Leith Dispensary.—The term Molluscum was introduced into cutaneous pathology by Dr. Willan, to designate a disease characterized by movable tumours on the skin, little sensible and often elastic to the touch, and not affecting the general health of the patient.

Professor Tilesius had undoubtedly described the disease in the case of a beggar at Muhlberg in 1793, but without giving it a name; and we are inclined to think with Dr. Jacobovics, of Pesth,* that Dr. Willan's term *molluscum* was more probably derived from Professor Tilesius's description, than, as Alibert and Biett think, from the resemblance of the tubercles to those on the bark of the maple tree.† The disease, however, which we are presently engaged with was not known to Dr. Willan, and indeed was not known to Dr. Bateman until after the publication of the second edition of his Synopsis, about the year 1814 or 1815. "A patient was sent to me," he says, "affected with a singular species of molluscum, which appears to be communicable by contact. The face and neck of this young woman were thickly studded with round prominent tubercles of various sizes, from that of a large pin's head to that of a small bean, which were hard, smooth, and shining on their surface, with a slight degree of transparency, and nearly of the colour of the skin, the tubercles were all sessile, upon a contracted base without any peduncle. From the larger ones a small quantity of milk-like fluid issued on pressure, from a minute aperture, such as might be made by a needle's point, and which only became visible on the exit of the fluid. The progress of their growth was very slow; for the first tubercle had appeared on the chin a twelve month ago, and only a few of them had attained a large size,—some of the latter had recently become inflamed, and were proceeding to a slow and curdly suppuration; and the cervical glands lying under those on the neck were also swollen and discoloured, as if proceeding to suppurate. She ascribed the origin of this disease to contact with the face of a child whom she nursed, on which a tubercle of the same sort existed; and, on a subsequent visit, she informed me, that two other children of the same family were disfigured by similar tubercles." "Since my attention," says Dr. Bateman, "was drawn to this species of tubercle, I have observed it in another, in an infant brought to me with *porrigo* (*impetigo*) *larvalis*, and, on investigation, it was found that she had apparently received it from an older child, who was in the habit of nursing it. In this case the milky fluid issued from the tubercles, and may be presumed to be the medium of the contagion."

Professor John Thomson shortly afterwards witnessed a series of cases in the Canongate of Edinburgh.

*March*, 1821.—"In a family resident in the Canongate of Edinburgh, there are three children, two boys and a girl, affected with *molluscum contagiosum.* About six months ago, small tubercles appeared on the face of the eldest boy, who, it is supposed, had caught the disease from some of his play-fellows, although none of them at present are known to have had it, nor has it been known ever to have existed in the neighbourhood. From this boy the disease was communicated to his sister, and to his little brother, a child of about nine months old, whom he occasionally carried about in his arms. The contagious nature of the disease is well evinced in the child. On the back of its hands a considerable number of tubercles are seen which have been produced by applying them to the face, and scratching those situated there during their inflammatory stage. Some of the tubercles are small, others large, some in a state of active inflammation, others nearly of the same colour as the skin, and quite free from pain. A few of them are pedunculated, but the greater of these number are attached by broad bases. They are seen on different parts

* Du Molluscum recherches critiques sur les formes la nature, et le traitement, par Dr. Jacobovics de Pesth. 1840.

† "Reinhardi visu fœdum corpus tectum est verrucis mollibus sive *molluscis*, et madidis sive myrmeciis."

of the face, on the forehead, eyelids, nose, lips, red of the lips, cheeks, and under the chin. Those under the chin have produced a considerable degree of inflammation of the skin, and tumefaction of the submaxillary glands. Two or three appear to be decaying, are shrunk and corrugated, and of a reddish brown hue. It is three months since the first appearance of the disease. The mother, though in the constant habit of nursing the youngest child, has not been infected."

Professor Thomson was consulted regarding the child of a farmer in the immediate vicinity of Edinburgh, who was affected with this disease in its characteristic form. It was traced to have been communicated to this child of the farmer's by a child of one of the farm servants; but this case could not be traced further. The farmer's child suffered severely from conjunctivitis, produced by the irritation of the tubercles on the edge of the eyelids. The disease was next communicated to the servant girl, who was in the habit of keeping the child during its illness, and appeared in its usual form on that side of the neck alone against which the child was in the habit of laying its face when affected with the ophthalmia. The above cases appear to me extremely interesting in so far as they point out in the most unequivocal manner the contagious nature of the disease.

The first case of this disease which I had an opportunity of witnessing occurred at the village of Newhaven in the month of December, 1840. The child, a girl about eighteen months old, extremely robust and active, and belonging to one of the cleanliest and best class of fisher people, had been affected with the eruption for the last three months. It was first observed in the neighbourhood of the mouth and nose, and it now occupies the same localities together with the lower eyelids, and a few thinly scattered over the cheeks and neck. The mother states, that, when it was first seen the tubercles had very much their present appearance. This child was nursed on one breast, and, although weaned, has the habit of still sucking it. The tubercles vary in size from that of a pin-head to a horse-bean,—the smaller ones having very much the white opaque appearance of pearly granulations, the larger ones being a little more coloured. The smaller ones are round, the larger ones oblong and irregular in shape, very much resembling that of a horse-bean. They are sessile on a contracted base, not pediculated. The larger ones only emit a whitish fluid when pressed. They seem to be not the slightest source of uneasiness to the child, and do not even appear painful when pretty roughly handled.

This child communicated the disease to the breast of the mother, and it appeared entirely confined to the sebaceous glands around the nipple of that breast, which the child continued to suck.

The tumours on the breast are of various sizes, from that of a pea to a hazlenut, three of the larger ones being clustered together, all exude a thick whitish matter when pressed between the fingers, and they seem to be equally insensible to the touch as those on the child. They first appeared on the breast about a month and a half after those on the face of the child.

The largest of these tumours laterally became inflamed and extremely troublesome, from the irritation of the rubbing of the clothes against them.

Particular inquiry was made as to any other members of the family being affected with a similar eruption; but no trace of it could be discovered, and an attempt to find out the source of contagion to the child proved equally unsuccessful; indeed, from the inquiries made, had any similar case existed in the village, it must have been discovered.

*Treatment.*—Mrs. C., the mother, was anxious that something should be done for the tumours, as they afforded her considerable inconvenience from rubbing against her dress, and, at the suggestion of Professor Simpson, the tops of them were touched with caustic potass. The application afforded little uneasiness to the patient; the escharotic destroyed a portion of the tumours, and the remainder soon sloughed off by their bases, leaving a healthy granulating surface, which healed kindly, and no return of them took place.

As the child's health was not in the slightest affected, no treatment whatever was had recourse to. The tumours as they enlarged, generally suppurated, scabbed, and then fell off by their base, and as this happened to more of them than was generated, a decided diminution soon took place, and at the present time there are only a very few remaining.

Case II. presented itself at the Leith Dispensary for consultation on the 2d of April last. The child, Ann M'Queen, 2 years old, strong and healthy, has been affected with the disease for the last two months. The mother ascribes it to her having been carried about by a girl who had some "similar lumps" upon her body, while they resided at Dundee, and immediately before they came to Leith. The eruption at present occupies the left side of the neck and shoulder, and a few are scattered here and there upon the same side of the face and trunk of the body. The disease resembles very much in appearance the case last described. The small tumours have the same pearly appearance, and the larger ones, being slightly redder than the skin, and exuding a milky fluid from the orifice at their apex. The tubercles at present appear in groups, and irregularly scattered over the surface of the skin; their number may be from thirty to forty on the present patient. The mother states that the girl, who was primarily affected at Dundee, used to carry this child chiefly against that side of her neck. But neither the mother nor any other of the children in the family have any appearance whatever of the eruption. This, however, may be partly accounted for by the fact, that the dress of this child being tied up round the neck, prevents, in a great measure, at least, any immediate contact between the eruption and the skin of the other children. Several of the largest of these tumours were cut off with a pair of scissors, and the skin healed well afterwards; others were destroyed with the caustic potass and nitrate of silver, but still the number of them on the body of the patient is not much diminished.

The next case which presented itself was that of a young married man, whose wife I had attended in labour some weeks previous. It was observed during the progress of the labour, that numerous small tumours existed at the orifice of the vagina, and in the neighbourhood of the vulva, but, thinking that they might be condylomata, or warts, no further attention was paid to them. The husband, however, showed me a number of tumours on the penis, which bore the characteristic marks of *molluscum contagiosum*. Upon inquiry regarding similar tumours on his wife, he informed me that they were of the same kind as those on his penis. They occasioned him considerable annoyance, and he applied for the purpose of getting them removed. The larger ones were cut off with the scissors, and the smaller touched with nitrate of silver, and they have all entirely disappeared.

Since the above cases occurred, I have had an opportunity of witnessing a beautiful and well-marked case of this rare disease, occurring in a child under Dr. Henderson's care in the Royal Infirmary.

This disease is as yet to be regarded as entirely British,—the latest authors in France, Germany, and America, making no mention whatever of it in their respective countries. We find Rayer, indeed, who wrote in 1827, and who is for referring the molluscum of Bateman to disease of the sebaceous follicles, saying, "etaient ils autre chose que des tumeurs folliculeuses? est-il bien démontré que cette affection soit réellement contagieuse?" It would appear from the cases which we have quoted, and from those which we have ourselves observed, that the disease has now been seen sufficiently often to enable us to answer M. Rayer's question in the affirmative. Dr. Craigie, many years ago, saw reason to infer, from a case of the chronic molluscum which he communicated to the London Medico-Chirurgical Society, that this disease arises from "some morbid or vitiated state of the sebaceous follicles;*" and we have strong grounds for referring the contagious species with M. Rayer to disease of the sebaceous glands. The drawing of enlarged sebaceous follicles in M. Rayer's work on Diseases of the Skin is sufficient to show this, and resembles very much, indeed, the species of molluscum at present under consideration. Besides this fact, however, the position of *molluscum contagiosum* is principally that where the sebaceous glands are most numerous, as at the angles of the nose, mouth, and eyes; and in several attempts which we have made to inoculate the white matter of molluscum into healthy skin, they have all been attended with failure; while in one attempt which was made about a week previous to the period I write this, a slight enlargement is taking place over the follicle already, which leads to the suspicion that a tubercle is about to form on the spot. Nothing as yet, however, can be spoken positively of this observation.

*Structure of the Tumours of Contagious Molluscum.*—Dr. Bateman having mentioned that the milky fluid which these tumours exude seems to be the medium of contagion in this disease, it appeared that it might be of advantage to examine the appearance of the milky discharge, and the structure of the tumour under the microscope. For this purpose, one or two of them were removed from the skin of Case II. on the occasion of her presenting herself at the Leith Dispensary on the 2d of April. The same afternoon, they were examined by my friend, Professor Reid, and myself, under his microscope, and a structure which we were unacquainted with, and which appeared to us peculiar, was noticed. Some days afterwards I removed one from Case I., which exhibited the same appearances; and, more lately, the tumours from the penis of Case III. presented analogous characteristics. Since then, I have had many opportunities of examining these tumours, taken from Case II., and more lately, through the kindness of Professor Allen Thomson, under his very powerful microscope.

The structure of the tumours is that of numerous cells, which secrete a whitish milky fluid. This is received from the cells into a central cavity, or irregular-shaped canal, in the interior of the tumour, which conveys the secretion to the orifice of it, where it exudes spontaneously, or on the application of pressure. The cells of which tumours are composed, when examined after the removal of the skin and cellular tissue, and while they are still covered with their investing membrane, have an irregular quadrilateral shape, some being five-sided, and presenting a very similar appearance to the cells of a honey-comb. When a thin section, however, is made of the tumours, and placed under the microscope, they are seen to have a long irregular shape, their external extremity being in contact with the investing membrane of the tumour, their internal one being prolonged into a nipple-like prominence. At the same time they are observed to be filled up with the peculiar globules of which the milky fluid is composed. Towards the external extremity of the cells, however, the globules are observed to be less distinct than at the internal

*Craigie's Pathological Anatomy, p. 643.

extremity. When the peculiar milky fluid is placed under the microscope, it is observed to be entirely composed of nucleated cells. These are very irregular in their shape, some being oblong, others ovate, and others having one side straight and the other of an irregular oval. They vary also a little in size. They are found to be about 1000th part of an inch in size, being, consequently, two and a-half or three times the size of pus globules, and three and a-half times as large as those of blood. This body will be observed to consist of an external, thin, and transparent membranous vesicle, somewhat flattened in its shape, and when these bodies are massed together serving to unite them ; and of an external vesicle, generally filled with small granular matter. This granular mass, in some, filled completely the tunic vesicle ; in others, it receded from its inner wall. These nucleated granules have, in fact, some resemblance to those of the epithelium. Some of the granules do not possess the external vesicle, and others possess it of a larger size. This Professor Thomson is inclined to attribute to the more or less mature state of the secretions. The external vesicle not being yet developed until it reaches very nearly the free surface of the secreting cells, in this ways the smaller size of the vesicles towards the outer extremity of the secreting cell, and their large size towards its internal or mamillary extremity, is easily accounted for—the external scale or vesicle not being at first developed, but its development and increase in size taking place as it becomes fit for evolution. In this way we find that the formation of this diseased structure is very similar to the development of animal and vegetable tissue, and not unlike that which Müller has described as taking place from the development of some kinds of carcinomatous structure.

The manner in which this disease must be communicated resembles much the account which Schwann and Schleiden have given of the development of animal and vegetable tissue.* The nuclei protrude young cellules, which project from their surface as the watch-glass from the watch. As growth proceeds, the young cell increases in size, while the nucleus remains imbedded in its wall. "Fresh nuclei form within the cavity of these young cells, and from a repetition of this process result successive generations of cells. The walls of the young cells are perfectly transparent, but those of the older cells become thickened, and in animal tissues often converted more or less into a fibrous structure." When one of these peculiar granules of which the milky fluid of this disease is composed, enters a sebaceous follicle, a similar process to that just described of nucleolar increase and cellular development take place,—the external vesicle of the primitive cell soon assuming a fibrous structure, and constituting the investing membrane of the subsequent tumours.

The peculiar cells of which the white fluid is composed are easily distinguished by a very moderate magnifying power, and appear to be peculiar to this disease, in so far as I have been able to observe. With this view I have observed microscopically the contents of atheromatous and melliceritious tumours, the contents of sebaceous follicles in health and in disease, &c., but have not been able to see anything similar. As a proof of their being easily distinguished again under the microscope, I found a small abscess at the base of a cluster of these tumours on the neck of one of the patients. I took some of the contents of it, for the purpose of examining whether or not it might be the same secretion as the molluscum; but it was found entirely composed of pus globules. The milky fluid of the molluscum was then mixed with it, together with some blood globules, and the vesicles of molluscum, being much larger than either of the other two, were easily distinguished ; and, although the magnifying power was not more than 100 diameters, the distinction between the three sets of globules was easily made out.

*General Observations.*—We have already mentioned that this disease appears to be entirely British ; no case of it having as yet been recorded in so far as we are at present acquainted in the records of any other country. It will, doubtless, however, be sooner or later noticed in other countries.

It would also appear to be entirely an infantile disease ; at least primarily, by which I mean that the first of a series of cases (the aboriginal one) has always been an infant. The first case that Dr. Bateman saw, that of a young woman, received the contagion from a child affected with the disease, whom she nursed ; two other children of the same family were also affected with it. The second case was also that of an infant who had apparently got it from an older child who was in the habit of nursing it. The series of cases which Cazenave and Schedel have related, and which occurred to Drs. Carswell and Thomson, first appeared on a young boy, next upon a girl, and then the infant at the breast.

The case which has been related on the authority of Professor Thomson, and which occurred in the country, originally appeared in a child of one of the farm-servants. This child communicated it to a child of the farmer's, it being thus conveyed by the most direct contact to the neck of the servant girl who kept the last mentioned child.

Dr. Henderson's case, where, I believe, he could not trace the contagion, also occurred in a child, and the two first series of cases which have been here related occurred in children.—The first case appeared on a child, and was

* On the nature and structural characteristics of morbid growths, by J. Muller, M. D., translated by C. West, M. D. 1840, p. 21.

communicated to the breast of the mother. The second case was communicated to the child by an older child that nursed her. The third case which I have related cannot be adduced either in evidence for or against, as, from delicacy, it was impossible to gain any satisfactory information from the parties. We have every reason, therefore, for believing that it is a disease, if not entirely originating in children, as certainly most frequently originating in, and being communicated to children, and therefore to be looked upon as truly an infantile disease.

*Character of the Eruption.*—The eruption in all the four cases which I have witnessed has borne a striking resemblance to each other. They all commenced in the shape of minute pearly granulations, and, increasing in size, became more of the natural colour of the skin. When of the size of a vetch, a minute opening generally became visible on their apices, and emitted a whitish milky fluid on pressure; as the tumours enlarged, they became irregular in shape and "sessile upon a contracted base." Their size varied from a pin-head to a hazel-nut, and their progress in reaching the largest size was very various, but generally slow. In Bateman's case a twelvemonth had elapsed between the period at which the tubercles begun, and that at which the application was made.

In Case II. they followed a much more rapid course than in any of the other cases. They may be looked upon as most generally occurring in irregular clusters of 3, 5, and sometimes even 20. This character is to be distinctly noticed in Dr. Bateman's plate, and is beautifully pourtrayed in a delineation of the first of Dr. Thomson's cases, which exists in his magnificent collection. In all the cases we have seen, this fact has been noticed. Altho' they bear a very chronic character, they appear to undergo spontaneous destruction and cure.

When this takes place, either a slow suppuration is established, which soon leads to the shrinking up and death of the tumours and their subsequent falling off; or, as we have seen when they were frequently irritated by picking them with the fingers, inflammation was excited, which soon led to the shrinking up and death of the tumours before any suppuration was established.

The *Molluscum contagiosum* appears not to be a disease of a dangerous nature. The case which Cazenave and Schedel relate on the authority of Dr. Carswell, proved fatal, but whether from this disease or any other is not stated. The case which lately occurred in the Royal Infirmary, under the care of Dr. Henderson, proved fatal; but we believe that death did not take place from the cutaneous disease. All the other cases which have been recorded were of an extremely mild nature, and, indeed, unless from the irritation of the tumours in the neighbourhood of the eyelids and mouth, giving rise to conjunctivitis and slight irritative fevers; the disease might be said to be one without even uneasiness. Sometimes, however, we find that the conjunctivitis proves very distressing. Professor Thomson informs me that the farmer's child, which has been previously mentioned in his second series of cases ran great danger of losing its eye-sight in consequence. Another annoying affection which has been mentioned by Dr. Bateman, and occurred to a considerable extent in Professor Thomson's and Dr. Carswell's case, was an enlargement of the glands in the neighbourhood of the eruption. This latter is very likely to occur in consequence of the irritation of the eruptive disease on the skin in their immediate neighbourhood.

*Treatment.*—We have already mentioned the course which nature follows in getting rid of these tumours, and in one case we allowed her to keep the treatment in her own hands till nearly a perfect cure was established.

In other cases, however, their progress is slow, troublesome, and they disfigure the person; it is, therefore, necessary to do something for their removal. Professor Thomson found in his experience the sulphate of copper, both in strong solution, and applied solid, effected the destruction of the tumours with sufficient rapidity. Previous to my being aware of the observations of Professor Thomson, I had applied the solid nitrate of silver to the tops of the tumours, and in one case I used the caustic potass, which certainly proved by far the most speedy and effectual method of cure. From the insensibility of the tumour the application of escharotics gives rise to no pain, and their destruction is speedily effected.

We have not given a proper trial to internal remedies in this disease. Dr. Bateman, however, found in his first case, that, after administering the arsenical solution in small doses for a month, "the tubercles were universally diminished, both in number and magnitude, most of them having gradually subsided, a few, especially on the neck, had suppurated." We have tried the arsenical solution in small doses; in Case II., it was not, however, continued so long. We also tried the *Aq. Potassæ*, and found no appearance of improvement whatever, although both sets of remedies were continued for about a fortnight. Indeed, during their administration, the disease seemed to follow its usual course of production and of spontaneous suppuration and decay. In Case I. also, the disease followed a very similar course to what Dr. Bateman has described, and has become nearly free of the affection, although no treatment whatever has been had recourse to. With regard to other remedies, we have made no trial whatever of them, and are inclined to look upon internal remedies in general as too tedious, when the local ones can be applied with so little pain to the patient, such surety to the destruction of the tumours, and in so much a shorter space of time.

*Ed. Med. and Surg. Journ.*

